# Experiences with interferon-free hepatitis C therapies: addressing barriers to adherence and optimizing treatment outcomes

**DOI:** 10.1186/s12913-019-3904-9

**Published:** 2019-02-01

**Authors:** Avy A. Skolnik, Amanda Noska, Vera Yakovchenko, Jack Tsai, Natalie Jones, Allen L. Gifford, D. Keith McInnes

**Affiliations:** 1 Center for Healthcare Organization and Implementation Research (CHOIR), Edith Nourse Rogers VA Medical Center, 200 Springs Road (152), Bedford, MA 01730 USA; 2 Department of Health Law, Policy, and Management, Boston University School of Public Health, 715 Albany Street, Boston, MA 02118 USA; 3 Division of Infectious Diseases, Providence VA Medical Center, 830 Chalkstone Ave, Providence, RI 02908 USA; 4 Veterans Affairs (VA) New England Mental Illness Research, Education, and Clinical Center (MIRECC), 950 Campbell Ave, West Haven, CT 06516 USA; 5 Department of Psychiatry, Yale University School of Medicine, 333 Cedar Street, New Haven, CT 06511 USA; 6 University Health Services, University of Massachusetts, 150 Infirmary Way, Amherst, MA 01003 USA; 7 Alpert Medical School, Brown University, 222 Richmond St, Providence, RI 02903 USA

**Keywords:** Hepatitis C, Veterans, Adherence, Health behaviors, Qualitative methods

## Abstract

**Background:**

Millions of Americans are living with hepatitis C, the leading cause of liver disease in the United States. Medication treatment can cure hepatitis C. We sought to understand factors that contribute to hepatitis C treatment completion from the perspectives of patients and providers.

**Methods:**

We conducted semi-structured interviews at three Veterans Affairs Medical Centers. Patients were asked about their experiences with hepatitis C treatments and perspectives on care. Providers were asked about observations regarding patient responses to medications and perspectives about factors resulting in treatment completion. Transcripts were analyzed using a grounded thematic approach—an inductive analysis that lets themes emerge from the data.

**Results:**

Contributors to treatment completion included Experience with Older Treatments, Hope for Improvement, Symptom Relief, Tailored Organized Routines, and Positive Patient-Provider Relationship. Corresponding barriers also emerged, including pill burden and skepticism about treatment effectiveness and safety.

**Conclusion:**

Despite the improved side-effect profile of newer HCV medications, multiple barriers to treatment completion remain. However, providers and patients were able to identify avenues for addressing such barriers.

## Background

Approximately 4 million people in the United States are living with the hepatitis C virus (HCV), the most common blood-borne infection and the leading cause of liver disease in the U.S [1–3]. The goal of treatment is to achieve sustained virologic response (SVR), defined as an undetected HCV lab result 12 weeks after the course of treatment has been completed. Many people living with HCV remain untreated, largely because the disease progresses slowly, extra-hepatic symptoms are subtle, and many living with HCV are asymptomatic [3]. Seeking HCV treatment may be further hindered by concerns about medication side effects given previous interferon-based HCV treatment regimens, which caused severe adverse reactions, including flu-like symptoms, fatigue, severe depression, and suicidal ideation [4]. These older medications were not only less effective compared to newer antivirals, but side effects made adherence difficult and SVR challenging to achieve. Newer combination directly-acting antiviral (DAA) therapies such as sofosbuvir/ledipasvir, among others, are not only more effective, they are also associated with less severe side effects [5]. These newer medications however are quite costly, still carry side effects, often involve more complex dosing schedules, and carry the potential to develop viral resistance, rendering the medications ineffective [6, 7]. Because the duration of therapy is shorter than previous therapies, each dose is more important and near-perfect adherence is critical.

While literature exists regarding adherence-promoting factors for interferon-based treatments, few have examined adherence promoting factors for newer medications. Understanding such factors is critical because individuals living with HCV often present with multiple psychosocial challenges that can make maintaining appointments and adhering to treatment recommendations difficult [8]. This is especially true for veterans with HCV, who are over-represented in the population of individuals living with HCV [9] and who are often contending with co-occurring substance use disorders, depression, anxiety, posttraumatic stress disorder (PTSD), homelessness, and other socio-economic challenges [8–10]. Thus, while newer HCV treatments are better-tolerated, these challenges can still threaten treatment adherence.

In 2015 there were an estimated 6% of veterans, over 200,000, in Department of Veterans Affairs (VA) care who were HCV antibody-positive, making the VA one of the largest providers of HCV care in the U.S [11, 12]. With the newer medications, there has been a concerted effort in VA to rapidly treat all cases, with 106,000 treated between 2014 and early 2018 [12]. As such, the VA presents an ideal setting to study adherence and treatment completion facilitators. We sought to investigate veteran and provider experiences and perspectives with newer, all-oral, interferon-free HCV medications, with particular interest in the factors that contribute to and interfere with, successful completion of treatment.

## Methods

We conducted a qualitative study using semi-structured interviews with providers and patients at three VA medical centers in the New England region. We explored patient and provider experiences with HCV treatments, perceptions and beliefs regarding treatment effectiveness, and barriers and facilitators of HCV treatment completion.

### Participants

All providers working within HCV clinics at each of the three sites were invited to participate. Veterans were identified using a master list of patients enrolled in the three clinics from 2014 to 2016. A random selection of patients who had initiated or completed HCV treatment with newer, interferon-free medications were invited to participate. Study team contacted patients by phone and patients indicating interest were mailed an informational sheet explaining consent. Participants were subsequently interviewed either by phone or in person. Verbal consent was obtained from participants before interviews commenced. The project and its procedures were considered part of a VA quality improvement project, which was deemed exempt from the Bedford, Providence and West Haven medical centers’ Institutional Review Boards in accordance with VHA Handbook (1058.05) quality improvement guidelines.

### Data collection

Semi-structured interviews were conducted by study team members at the three New England VA medical centers (Sites A, B, and C) from October 2015 to May 2016. All interviews followed a semi-structured interview guide, were audio-recorded, and transcribed verbatim. Patient questions elicited perspectives on HCV, treatment regimens, adherence facilitators and barriers, quality of HCV care, and stigma associated with HCV. Providers were asked about perceptions of treatment adherence, barriers, and facilitators of the newer treatments.

### Data analysis

Transcripts were analyzed qualitatively using procedures derived from grounded theory methodology, a form of qualitative research which operates inductively, allowing themes to emerge from the data [13, 14]. First, we conducted open coding, a process in which concepts are identified within text fragments and assigned descriptive terms (codes). Three reviewers conducted open coding on two provider and three patient interviews, came to consensus on the open codes, and developed a code book. All codes were derived inductively. The remaining transcripts were divided among three reviewer pairs and coded using this codebook. Each transcript was coded by one primary and one secondary reviewer. Additional codes emerged during this phase and were added to the codebook, and transcripts were then re-reviewed for the new codes using constant comparison analysis [13]. In the next phase, themes were developed by merging some codes and refining others. The team met regularly to discuss and refine themes, and to compare themes across sites and participant type.

## Results

Of the 66 patients contacted, 28 did not respond or declined to participate. Of the 15 providers contacted, five declined to participate. Providers included five physicians, three clinical pharmacists, one nurse practitioner, and one RN nurse. All patients had confirmed HCV infection. Sixteen patients had prior treatment experience with PEGylated-interferon- or interferon-based regimens, while 22 were treatment-naïve. Sites A, B, and C included, respectively, 20 interviews (17 patients, 3 providers), 16 interviews (11 patients, 5 providers), and 12 interviews (10 patients, 2 providers). Table 1 describes patient characteristics.

**Table 1:** Patient Characteristics

Characteristic (*N* = 38)	N (%)
Age	
40–49	2 (5)
50–59	9 (24)
60–69	24 (63)
70≤	3 (8)
Sex	
Male	33 (87)
Female	5 (13)
Race	
White	25 (66)
Black	9 (24)
Missing or Other	4 (10)
Education	
High school or less	16 (42)
Some College or more	19 (50)
Missing/unknown	3 (8)
Income	
< $30,000	14 (37)
> $30,000	8 (21)
Missing/unknown	7 (18)
On disability	9 (24)
Any homeless history	14 (37)
Any mental health or substance use disorder diagnosis	28 (74)
Years since initial hepatitis C diagnosis	
< 5	5 (13)
5–10	5 (13)
11–20	9 (24)
21–30	9 (24)
Unknown	10 (26)
Previous hepatitis C Treatment	
Treatment experienced	16 (42)
Treatment naïve	22 (58)
Sustained viral response	
Yes	21 (55)
No	1 (3)
Not yet known	16 (42)

Five themes emerged from patient interviews and three of them also emerged in provider interviews. All treatment facilitators described by providers were corroborated by patients. The facilitators contributed to treatment completion in three ways: increasing the patient’s willingness to initiate treatment, improving adherence to medication, or decreasing missed appointments. These themes are presented below and in Table 2.

**Table 2:** Themes, Definitions, and Patient Quotations

Facilitators	Definition	Quotations
Experience with Older Treatments	Perceptions of current HCV treatments and their side effects compared with prior HCV treatment experience when applicable.	“I feel a little tired, but that’s it. Oh, and a rash -nothing serious, just itch. With the Interferon treatment, like I said, I had shortness of breath going up stairs, irritable, definitely irritable. So compared to the interferon, this is a walk in the park.” – *Veteran, Treatment-experienced, Site B* “Also when some of the side effects started coming to the surface, you know, I was told to my face that that wasn’t from the (new HCV) medication, and it is. There’s no doubt. It has been.” *– Veteran, Treatment-naïve, Site A*
Symptom Relief	Participant perceives amelioration of HCV-related symptoms that they attribute to initiating or completing treatment.	“Absolutely unbelievable. Like I can’t stress enough, you don’t realize that this disease drags you down. Really, when you get this disease and you keep it for that length of time, it is wearing on you, you just don’t realize it. I think you get used to the symptoms. It takes so long to develop them that you get used to it, and I don’t think anybody notices it until you take the cure. And then it’s: Whoa! I felt awful all those years and never knew it. It’s just an unbelievable difference.” *– Veteran, Site A*
Hope for Improvement	Participant expresses optimism regarding SVR, and/or possibility of improved health or longevity of life, such that they feel motivated to complete treatment.	“Yeah, I think so. I think that they’re finally offering hope. I’ve heard of several different people at the VA who’ve been cleared, and that’s what gave me hope that I could be clear, too.” *– Veteran, Site C* (I: Have you gained relief?) “Well *mentally* I have – I have mental relief, because before, I didn’t feel like there was hope.” – *Veteran, Site A*
Positive Patient-Provider Relationship	Participant describes trust in provider, empathy, time taken to educate patient, or support contributing to treatment completion.	“(HCV doctor) is the best. She ended up hugging me, we high fived. She’s known how I’ve struggled with taking the treatments, and it was like she was happy for me too.” *– Veteran, Site C*
Tailored Organized Routines	Descriptions of consistent, re-curing habits or routines that contributed to the treatment completion. Includes social support, calendars, technology, and other strategies for medication adherence or appointments.	“I had a friend who called me every morning and said, ‘Take your medicine.’” *– Veteran, Site B* “I just have that in my schedule, because I take one other pill a day for my blood pressure, so I do that at the same time.” *– Veteran, Site A* “I have an alarm on my phone that rings every night at 11.” *– Veteran, Site C*

### Experience with interferon

Prior experience with older HCV treatments emerged as facilitator of treatment completion. Treatment-experienced veterans compared their prior treatment with the newer medications, with overwhelmingly positive perceptions of the latter. “Compared with interferon, the new medication…it was like the difference between night and day. There was hardly any side effects. I mean, it was just totally different.” Providers also observed that *treatment-experienced* patients viewed their *side effects as minimal*: “I think it’s easier for those who’ve been through interferon and ribavirin who now are on some of the newer drugs. You ask them about side effects, and they’re like, ‘Nothing.’ They’ve got nothing to report.” Providers observed, however, that treatment-experienced patients sometimes needed additional counseling, encouragement, and reassurance to try the newer medications, and to accept that the side effects would be unlike older regimens.

Conversely, *treatment-naïve* veterans more *frequently reported side effects* with the newer regimens and difficulty tolerating them: “It (new medications) made me lose 20 pounds, it made me sick, I couldn’t eat, it just was really…I would never do it again and I wouldn’t advise anyone to do it.” Providers also observed this distinction: “The people who have been treated in the past come in and say, ‘I feel great, I don’t feel any side effects, is this medication even working?’ And then I have patients who’ve never been treated before, and they’re all, ‘I’m so tired, I’m so fatigued.’” Overall, treatment-naïve patients perceived side effects as more pronounced than did those who had experienced interferon, perhaps because the latter were bracing for serious adverse reactions. Thus, it appeared that experience with older HCV medications could be a barrier to treatment initiation, but could later become a facilitator of treatment completion by contributing to ease of adherence.

### Hope for improvement

Hope facilitated treatment completion, with many veterans expressing *optimism about the prospect of being cured* and *extending their lives*. One veteran described how the treatment had affected his health: “Number one, you know you’re better. And number two, you know, *mentally* it gives you a boost thinking, ‘Gosh. I got a second chance on this.’” Other patients initially worried that treatment would fail but after receiving their initial “undetected” test result, their hope was renewed: “I didn’t know if it was going to work. Knowing my freaking luck, I’d be the one percent it don’t work on, you know? But to find out definitely it is working, I was walking on clouds there for a while.” This hope provided additional motivation that may have contributed to adherence. One patient who achieved SVR noted: “I was willing to do anything. I was sober, I wanted to live long enough to see my kids grow up.”

Conversely, a few participants expressed *concerns about the new treatments’ safety and efficacy*. “This is a fairly new medication I guess and with that you know, I wonder what can happen later on. Can I get cancer from this?” Similarly, an African American patient wondered whether there would be racial disparities in SVR rates with new medications, as was the case for interferon-based regimens [15] Interestingly, these contrasting emotions of hope and concern did not emerge in provider interviews.

### Symptom relief

Some veterans perceived improvement in symptoms after initiating HCV treatment. However, patients often expressed that HCV -related symptoms were so subtle, they went largely unnoticed until HCV treatment was initiated, at which point the patient noticed relief from a given symptom. Fatigue, for example, was a common symptom and several patients only became aware of how it had affected them after starting treatment. “But I *did* feel something because after I did the (treatment), I mean I was like a – I’m a different person. I feel fantastic. I was sluggish before, I was like – you don’t realize how (HCV) works on you until you don’t have it.” Some veterans described euphoria during treatment: “I felt like Superman. I felt terrific on it. I had this euphoric sense of I could do anything.”

Conversely, some veterans noted that a *lack of noticeable HCV symptoms* may have made initiating or remaining on treatment difficult or less of a priority: “I don’t know if I initiated (conversation about HCV treatment), or if my doctor initiated it. She probably did, because I could care less, you know? I had basically no symptoms. I’m not sick from it.” Providers, however, did not comment on symptom-relief. Thus it appears patients’ perceived lack of symptoms could create a barrier to treatment initiation, but once this barrier was overcome, noticeable symptom relief during treatment facilitated completion.

### Positive relationships with providers

Patients emphasized positive relationships with providers as being integral to treatment success, noting their sense of *trust*, experience of *consistent support, clear explanations of HCV and treatments, and provider efforts to decrease shame and stigma.* Some veterans discussed that knowing that their providers believed in their ability to complete treatment increased their *self-efficacy*. As one veteran noted, “I wanted to beat this thing. I knew I could beat it because my doctor, she was so adamant, you know? And so I knew I could do it.” Other patients sensed that their providers truly cared for them. “Well, the clinic itself I would say is fair, but if it wasn’t for (the two clinic pharmacists), if it wasn’t for them two, I don’t know. You know, they kind of kept me focused. And they kept my spirits and my morale going.” Other veterans appreciated providers who carefully explained the treatment process: “They always took their time, they always talked to me, they always explained what was going on.”

While providers did not directly comment on their relationship’s contribution to treatment success, the manner in which they spoke of patients conveyed *empathy*: “Some of these patients, they’ve waited a long time and they’ve made big changes in their lives, and they really want the treatment to work. And we (providers) really want to see it work.” Thus, providers appear to have invested in developing and maintaining relationships with patients which likely improved treatment success by increasing patient treatment initiation, adherence, and appointment attendance.

### Tailored organized routines

Patients were asked to reflect on their experience adhering both to medications and to follow-up appointments. Perhaps not surprisingly, having a daily routine promoted adherence. Routines could be enhanced by *social support* and *reminders*. Specific routines varied significantly. Some participants found that *taking medications at the same time each day* or *working the HCV regimen into existing medication routines* was helpful: “I gotta take pills anyways for my heart, so I take my (HCV) pills and put ‘em in with my heart pills, so every time I took my heart pills, I take those.” Providers noted the importance of *planning for the unexpected*: “He had the pill bottle at home and then also a small container for travel that he took with him, because he never knew if he was going to be home in time.” Patients and providers described two factors helpful in enhancing this routine: 1) *use of alarms, calendars, and technology as reminders* and 2) use of *social support* for maintaining routines. “I really had to stay on top of the medication and all of that. And I had my wife help me, we wrote everything out on the calendar and made sure that I didn’t miss any appointments, and the medications- taking the medications at the same time.” Routines varied considerably by individual characteristics and experiences such as comfort with technology, self-knowing, family situation, and experience managing other chronic conditions.

Veterans and providers noted the significance of *pill burden*, which varied greatly based on provider decisions and the patient’s HCV acuity and genotype, prior treatment experience, and status of cirrhosis. Veterans commented on number and size of pills: “Well, with that medication, you had to take it every single day, and it was like six pills you had to take twice a day. And it wasn’t small pills either... Looked like the size for a horse to take.” One provider noted that for patients with cognitive impairments, even simpler regimens could create adherence challenges: “For him, I’m discovering that maybe just a once-a-day pill would’ve been a better choice, because remembering twice-a-day has even been a barrier. He’s even taken the pills out, and put ‘em on the counter, and walked away and forgotten them.”

## Discussion

### Discussion

In an era of great promise about the new interferon-free medications, interviews with 38 patients and 10 providers led to identification of several factors that facilitate or hinder treatment initiation, medication adherence, and reduced appointment no-shows. The five facilitators were Experience with Interferon, Hope for Improvement, Symptom Relief, Tailored Organized Routines, and Positive Relationships with Providers. Several factors known to complicate medication adherence did not emerge in this study, but still bear mentioning. First, the medications’ high cost, $84,000 to $159,000, may represent a barrier [16]. Patients in this study were enrolled in VA and thus bore little, if any, medication cost. Additionally, transportation, parking, clinic distance, and long wait times can undermine patient willingness to seek treatment or adhere to appointments [17]. Lastly, substance use disorders (SUDs) and mental illness have interfered with older, interferon-based treatments [16]. Yet in this study neither emerged as treatment adherence barriers. This finding is encouraging in that it suggests that SVR may be obtainable for a larger portion of HCV-infected persons living with SUDs, mental illness, or other psychosocial stressors. Our findings suggest steps that providers can take to improve patient care, the patient experience, and treatment outcomes.

### Practice implications

#### Consider patients’ previous HCV treatment experience when planning for treatment

A patient’s prior experience with the older HCV therapies was an important determinant of how they perceived the interferon-free medications. Treatment-naïve veterans embarking on interferon-free treatment seemed prone to side effects in our study, especially fatigue and headache, but also reporting nerve pain, stomach upset, and rash. Providers should consider that side effects may be related to the new HCV treatments, despite industry claims that there are few side effects. With treatment-naïve patients, providers should not downplay potential side effects, while with treatment-experienced patients, providers may want to emphasize that newer medications have a different and more favorable side effect profile. For patients reluctant to pursue treatment, providers may find it useful to discuss habituation of indolent symptoms related to HCV (e.g., fatigue) over time, and the possibility that these symptoms may improve with treatment. Establishing realistic but hopeful expectations for treatment contributes to the patient experience and patient-centered care, which are important but oftentimes overlooked aspects of treatment that should be considered along with achievement of SVR.

#### Recognize the role of hope

Interestingly, discussions about hope did not emerge in provider interviews, while patients placed hopes on the new medications for a cure and longer lifespan. Providers might ask their patients: “How might life be different if you clear the virus and are cured?” Providers’ encouragement of a patient’s hopeful attitude may contribute to improved treatment outcomes [18]. Addressing potential improvements in quality of life, symptom relief, reductions in the psychological burden of living with HCV, decreased risk of transmission to partners, and decreased mortality [19, 20] are all important topics in such a discussion. Patients previously treated with interferon-based regimens will benefit from information emphasizing the decreased side effects and, particularly for African Americans, the increased efficacy of the newer HCV medications [21].

#### Attend to the relationship

Patients consistently emphasized that a positive relationship with their provider promoted trust and enhanced feelings of self-efficacy, which have been identified as promoting health-seeking behavior and treatment adherence with HCV and other conditions [18, 22]. Patients noticed when providers took time to provide detailed information about the virus and treatment options, and showed empathy and caring. A patient must trust their provider to admit imperfect adherence or loss of medications without fear of being embarrassed or shamed. When providers empathetically inquire about missed doses, substance use, or other life circumstances, they are likely to obtain information that contributes to the treatment plan and more successful outcomes. This also reduces stigma, which inhibits healthcare seeking behavior [23].

#### Assist patients in developing tailored, organized medication routines

While patients’ life contexts differed significantly, treatment was optimized through daily, personalized routines. Providers should encourage routines and reminder systems, and also discuss with patients contingency plans for late or missed doses. Providers should become aware that for some newer HCV medications a translucent container (such as a daily pill box) can decrease potency; thus providers should inquire how their patients are storing pills. Providers should also discuss housing status, psychosocial stressors, work schedules, transportation, financial limitations, and social support because of their effects on treatment adherence. When feasible, involving patients’ social supports in treatment planning and discussing options for reminders (electronic or otherwise) can enhance both the development and implementation of routines.

## Conclusions

There are several study limitations. This study was conducted at three VA medical centers within one region of the U.S. It is possible that patients and providers from other regions may have perspectives not captured in this study. Nearly all patient participants were men. Additionally, the scope of the study did not enable interviewing veterans who either had initiated but then stopped interferon-free treatment, or who had considered this treatment but decided against it. Thus, there may be other barriers to treatment initiation and completion that we are unaware of. Patients outside the VA system might face real or perceived financial barriers to obtaining direct acting antivirals that were not addressed in this analysis and require further study. Finally, a limited number of providers participated and thus we may not have fully captured the range of HCV provider perspectives.

Despite medications with increased efficacy and effectiveness, and few onerous side-effects, there is much to be learned about achieving high rates of SVR while delivering positive patient treatment experiences. Newer HCV medications carry considerable financial costs, and are not without context-related adherence barriers, side effects, and potential for complex dosing. This suggest that even amidst optimism about dramatically reducing rates of HCV, patient access to medications, treatment adherence and treatment completion remain critical issues in combatting HCV. Our study has identified ways that providers can enhance the prospects for successful treatment. As indicated above, however, there are still gaps in our knowledge of the experience of patients, providers, and caregivers, with the newer medications. Additional studies in other HCV-infected populations (e.g. women, persons with mental illness), using other methods, such as survey research, would add to our understanding of treatment initiation and completion, and patients’ experience with HCV care.

## Abbreviations

DAA: Direct-Acting Antivirals
HCV: Hepatitis C Virus
PTSD: Post-Traumatic Stress Disorder
SUD: substance use disorder
SVR: sustained virologic response
VA: Department of Veterans Affairs
